# Mixed forest plantations can efficiently filter rainfall deposits of sulfur and chlorine in Western China

**DOI:** 10.1038/srep41680

**Published:** 2017-01-30

**Authors:** Hairong Zhao, Wanqin Yang, Fuzhong Wu, Bo Tan

**Affiliations:** 1 Key Laboratory of Ecological Forestry Engineering, Institute of Ecology and Forestry, Sichuan Agricultural University, Chengdu 611130, China

## Abstract

Forest filtering is a well-known and efficient method for diminishing atmospheric pollutant (such as SO_4_^2^^−^ and Cl^−^) inputs to soil and water; however, the filtering efficiencies of forests vary depending on the regional vegetation and climate. The rainy area of West China has suffered from heavy rainfall and human activity, which has potentially resulted in large amounts of sulfur and chlorine deposition, but little information is available regarding the filtering effects of typical plantations. Therefore, the migration of SO_4_^2^^−^ and Cl^−^ from rainfall to throughfall, stemflow and runoff were investigated in a camphor (*Cinnamomum camphora*) plantation, a cryptomeria (*Cryptomeria fortunei*) plantation and a mixed plantation in a 9-month forest hydrology experiment. The results indicated the following: (i) The total SO_4_^2^^−^ and Cl^−^ deposition was 43.05 kg ha^−1^ and 5.25 kg ha^−1^, respectively. (ii) The cover layer had the highest interception rate (60.08%), followed by the soil layer (16.02%) and canopy layer (12.85%). (iii) The mixed plantation resulted in the highest SO_4_^2^^−^ (37.23%) and Cl^−^ (51.91%) interception rates at the forest ecosystem scale, and the interception rate increased with increasing rainfall. These results indicate that mixed plantations can effectively filter SO_4_^2^^−^ and Cl^−^ in this area and in similar areas.

Rapid expansion of industrialization has resulted in the release of more and more sulfur (mainly as SO_2_) and chlorine (mainly as Cl_2_) into the atmosphere, which has resulted in an increasing number of health problems. Acid mist can directly intrude lungs and cause pulmonary edema, and the long life of acid mist in acidic environments has resulted in an increased probability of several diseases, such as atherosclerosis and myocardial infarction^1^. Among others, sulfur and chlorine are two of the main acid pollutants in the air, soil and water. On the one hand, acid pollutants influence aquatic biological communities, which can increase the dissolution of toxic metals in water and allow the metals to enter the food chain. On the other hand, acid pollutants inhibit organic matter decomposition and nitrogen fixation, which can lead to soil impoverishment and affect plant growth^2^,^3^. These processes will eventually accelerate species extinction and destroy ecological balance, causing serious consequences. According to the estimates of Streets *et al*.^4^,^5^,^6^, sulfur and chlorine emissions continue to increase. How to diminish sulfur and chlorine pollutants is an urgent problem. In comparison with chemical and physical methods, forest filtering is thought to be the most environment-friendly method for decreasing the concentrations of air pollutants such as sulfur and chlorine^7^,^8^. Atmospheric input and migration in a forest ecosystem during rainfall are often divided into the three following vertical levels: the canopy layer, cover layer and soil layer^9^ (Fig. 1). These dry and wet pollutants can be directly intercepted and absorbed by different layers, and can directly reduce the concentrations of the pollutants. Furthermore, soil can absorb pollutants indirectly through a series of physical and chemical process, which can effectively reduce pollution^10^.

The filtering effect of the forest is decided by multiple factors. First, the filtration efficiency is significantly different among different forest types, mainly due to the forest vegetation and soil. The composition of forest vegetation can change the precipitation potential energy and distribution of elements during rainfall processes^11^,^12^. Kimura^13^ noted that the rich component (e.g., arbour, shrub, herb and moss) is beneficial for holding and intercepting water. In addition, soil physical and chemical properties are important for intercepting, filtering and transferring elements, and element transfer mainly occurs through the exchange of equivalent anions and cations. Second, several studies have shown that SO_4_^2−^ and Cl^−^ leaching fluxes are lower in broadleaf forests than in coniferous forests; however, the concentrations of SO_4_^2−^ and Cl^−^ in broadleaf and coniferous forests are nearly equal^14^,^15^. These differences are related to the plant density, crown density, plant branches and leaves, LAI, bark roughness and plant growth; moreover, the forest interception rate is proportional to the above factors^16^,^17^. Third, the rainfall characteristics (e.g., rainfall, rainfall intensity, rainfall time, time interval) directly affect the forest interception rate^9^,^18^. The interception rate increases with increasing rainfall within limits and decreases as the rainfall intensity increases. Because the ability of a forest to absorb precipitation and elements is limited, no additional interception will occur once a critical value is reached^16^,^19^. Fourth, the combination of seasonal rainfall and plant phenology has a periodic influence on forest interception. Rainfall characteristics are different during the rainy and dry seasons, and plant growth follows the rules of the four seasons based on the biological characteristics and morphological changes of the plant (especially deciduous species and annual plants)^20^,^21^. Based on the above four reasons, we hypothesize that mixed forests have a better filtering effect than broad-leaved and coniferous plantations during rainfall.

The rainy area of West China is located at the western edge of the Sichuan Basin and receives more than 1200 mm of precipitation annually. The central region receives up to 1700 mm of precipitation annually. In this area, human activity is intense, and large amounts of SO_4_^2−^ and Cl^−^ are deposited by rainfall, which could result in large amounts of soil and water pollution. Plantations are distributed in areas with concentrated human activity where their role in filtering air pollution would be more significant. Broadleaf camphor (*Cinnamomum camphora*), coniferous cryptomeria (*Cryptomeria fortunei*) and secondary mixed plantations are the typical types of plantations in this area; however, corresponding research is lacking. Therefore, we selected the three typical plantations described above as the object of this research. This study is part of a large project that characterizes regional ecological security. Here, we compare the hydrological processes and ion input fluxes in three typical plantations and used statistical and mathematical modelling techniques to investigate the factors that control the hydrology and biogeochemistry of SO_4_^2−^ and Cl^−^ inputs (tree-level and plot-level basis). In addition, we quantify the contributions of mixed input and canopy exchange processes to net bulk precipitation, throughfall, stemflow, surface runoff and soil percolating water fluxes. The results can be expected to reduce environmental pollution (including air, water and soil pollution) and provide a basis for regional plantation management.

## Results

**Figure 2:**
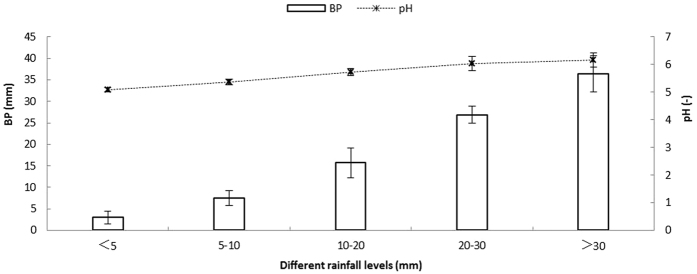
The relationship between pH and GP (grade of precipitation) under each mean BP (bulk precipitation) event.

**Figure 3:**
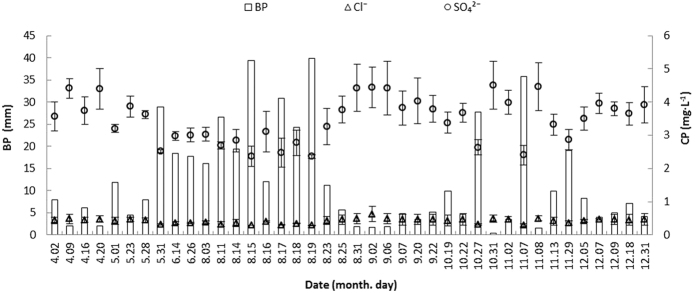
The relationship between CP (concentration of pollutants) and BP (bulk precipitation).

Bulk precipitation input. The total bulk precipitation was 492.72 mm over the 9-month input period, with single bulk precipitation events ranging from 0.39 to 39.90 mm. The pH significantly increased with bulk precipitation during the studied period (Fig. 2) and varied between 5.01 and 6.49, with a mean value of 5.53. Significant negative correlations were observed for the SO_4_^2−^ and Cl^−^ concentrations (Fig. 3), and the total deposition of SO_4_^2^^−^ and Cl^−^ was 43.05 kg ha^−1^and 5.25 kg ha^−1^, respectively. The flux of SO_4_^2−^ was generally 8.31-fold higher than the flux of Cl^−^, which resulted in relatively significant changes in the fluxes during the rainfall progress.

Chemistry of throughfall and stemflow. The famounts of both ions in the throughfall and stemflow in the three plots significantly decreased with increasing bulk precipitation. In contrast, the pH was positively correlated with bulk precipitation (Table 1). Both ion concentrations decreased due to interception by the canopy layer. Among the three plantations, the amounts of SO_4_^2−^ and Cl^−^ intercepted by the canopy layer were the greatest in the mixed plantation (13% and 10%), followed by the camphor plantation (9% and 7%) and the cryptomeria plantation (7% and 3%). When considering the mean pH of the bulk precipitation, the pH values of the throughfall and stemflow increased in the three plantations, and the greatest pH increase of 0.33 was observed in the stemflow.

**Figure 4:**
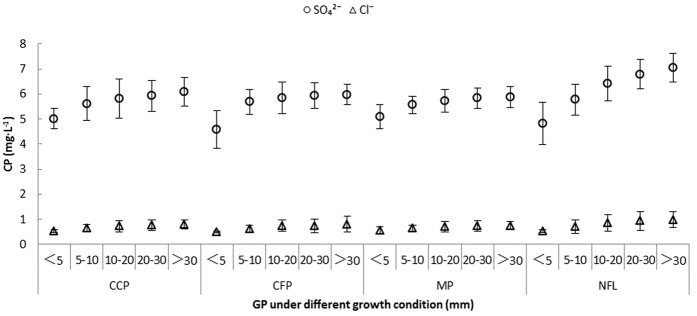
The relationship between CP (concentration of pollutants) and GP (grade of precipitation) in the SR (surface runoff water) under the four growth conditions. CCP: Camphor (*Cinnamomum camphora*) plantation, CFP: Cryptomeria (*Cryptomeria fortunei*) plantation, MP (mixed plantation) and NFL (non-forest land).

**Figure 5:**
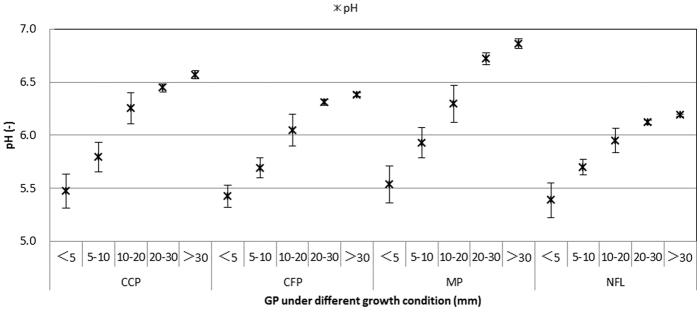
The relationship between pH and GP (grade of precipitation) in the SR (surface runoff water) under the four growth conditions. CCP: Camphor (*Cinnamomum camphora*) plantation, CFP: Cryptomeria (*Cryptomeria fortunei*) plantation, MP (mixed plantation) and NFL (non-forest land).

Chemistry of surface runoff. The total surface runoff outputs of SO_4_^2^^−^ and Cl^−^ in the non-forest land were 59.53 kg ha^−1^and 6.68 kg ha^−1^, respectively. Despite the unchanged sulfur and chlorine input rates that dominated the atmospheric supply of acidity, the concentrations of both ions significantly decreased in the surface runoff from the three plantations compared with the non-forest land (Fig. 4). The observed trends occurred among the three plantations, especially in the mixed plantation, and were explained by a 52% reduction in SO_4_^2−^ and a 60% reduction in Cl^−^ in the investigated area. In addition, the pH significantly increased with increasing precipitation (Fig. 5), and the pH of the surface runoff was the highest in the camphor plantation.

**Figure 6:**
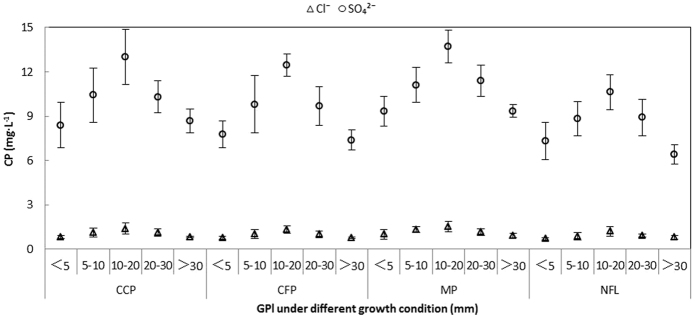
The relationship between CP (concentration of pollutants) and GP (grade of precipitation) in the SP (soil percolating water) under the four growth conditions. CCP: Camphor (*Cinnamomum camphora*) plantation, CFP: Cryptomeria (*Cryptomeria fortunei*) plantation, MP (mixed plantation) and NFL (non-forest land).

**Figure 7:**
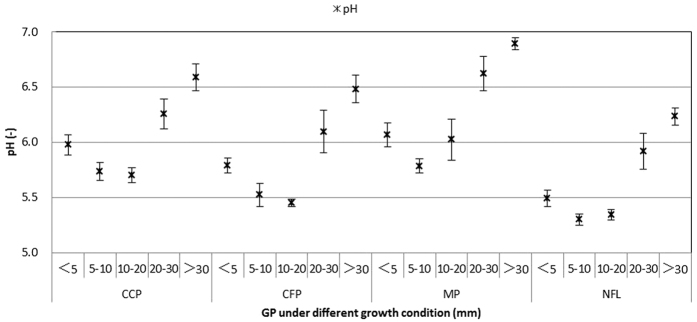
The relationship between CP (concentration of pollutants) and GP (grade of precipitation) in SP (soil percolating water) under the four growth conditions. CCP: Camphor (*Cinnamomum camphora*) plantation, CFP: Cryptomeria (*Cryptomeria fortunei*) plantation, MP (mixed plantation) and NFL (non-forest land).

Chemistry of soil percolating water. The total outputs of SO_4_^2^^−^ and Cl^−^ from the soil percolating water in the non-forest land were 55.86 kg ha^−1^and 7.66 kg ha^−1^, respectively. Both ion fluxes exhibited similar trends in the soil percolating water, and elevated fluxes of SO_4_^2−^ and Cl^−^ occurred in the 10–20 mm rainfall events (Fig. 6), which was usually associated with the presence of silt in the samples (not shown). The plantation with the highest SO_4_^2−^ interception rate in the soil layer was the camphor plantation (10%), followed by the cryptomeria (9%) and mixed plantations (4%). However, the interception rate of the soil layer for Cl^−^ was 10% in both pure plantations and 16% in the mixed plantation. In addition, compared with the non-forest land, the pH values increased in all three plantations (Fig. 7), and the pH of the soil percolating water was the highest in the mixed plantation.

**Figure 8:**
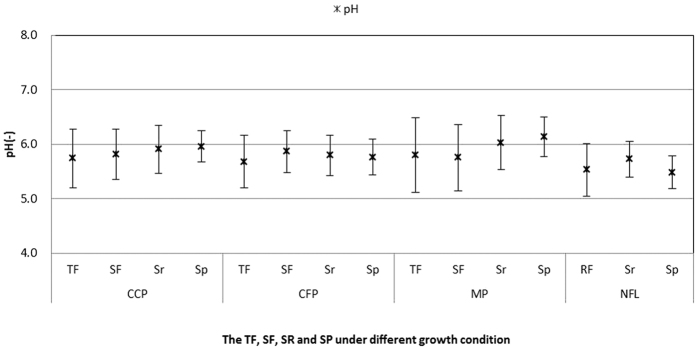
The mean values of TF (throughfall), SF (stemflow), SR (surface runoff) and SP (soil percolating water) under different growth conditions. CCP: Camphor (*Cinnamomum camphora*) plantation, CFP: Cryptomeria (*Cryptomeria fortunei*) plantation, MP (mixed plantation) and NFL (non-forest land).

Mean concentrations of SO_4_^2−^ and Cl^−^. Both ions followed a similar trend in the three plantation ecosystems. The soil percolating water had the highest mean pollutant concentrations, followed by surface runoff, throughfall, and stemflow (Table 2). Moreover, compared with the non-forest land, the concentrations of both SO_4_^2−^ and Cl^−^ decreased in the three plantations (except soil percolating water). In addition, the pH values hardly varied among the four plots, and the mean pH values in the three plantations were all higher than those in the non-forest land. The maximum-pH mean value was observed in the mixed plantation in the soil percolating water (6.13) (Fig. 8).

**Figure 9:**
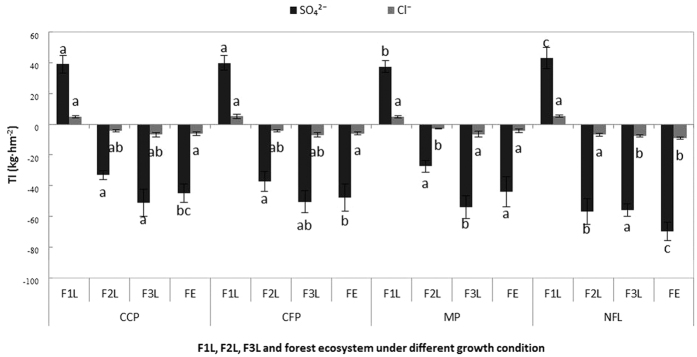
The TI (total input) of the F1L (forest canopy layer), F2L (forest cover layer), F3L (forest soil layer) and forest ecosystem (forest ecosystem) under four growth conditions. CCP: Camphor (*Cinnamomum camphora*) plantation, CFP: Cryptomeria (*Cryptomeria fortunei*) plantation, MP (mixed plantation) and NFL (non-forest land).

**Figure 10:**
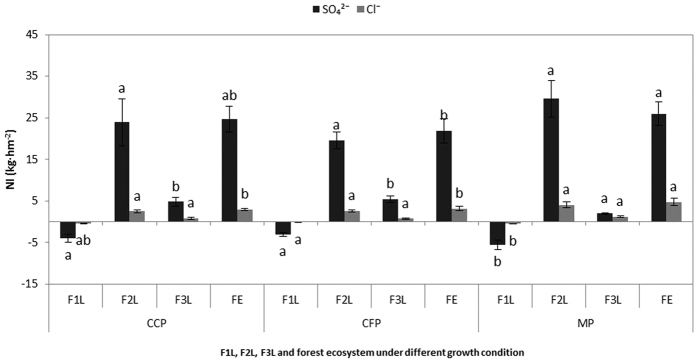
The NI (net input) of the F1L (forest canopy layer), F2L (forest cover layer), F3L (forest soil layer) and FE (forest ecosystem) in three plantations. CCP: Camphor (*Cinnamomum camphora*) plantation, CFP: Cryptomeria (*Cryptomeria fortunei*) plantation, MP (mixed plantation) and NFL (non-forest land).

The filtration of the three plantations for SO_4_^2−^ and Cl^−^. All of the inputs and outputs are shown in Fig. 9. On the one hand, the interception rates of SO_4_^2−^ and Cl^−^ were significantly different between the canopy layers, however, the interception of Cl^−−^ was very low, and the calculated interceptions of the canopy layers were very sensitive to the forest types. On the other hand, compared with the canopy layer, the interception rates of both ions by the cover layer and soil layer were high. However, this difference was not significant in most cases, except for the interception of SO_4_^2−^ by the soil layer. The final analysis concluded that the highest net input and interception rate of SO_4_^2−^ occurred in the mixed plantation (25.96 kg ha^−1^ and 37%), followed by the camphor plantation (21.86 kg ha^−1^ and 31%) and the cryptomeria plantation (24.71 kg ha^−1^ and 35%). Furthermore, the net input and interception rate of Cl^−^ was highest in the mixed plantation (4.72 kg ha^−1^ and 52%), followed by the cryptomeria plantation (3.13 kg ha^−1^ and 34%) and the camphor plantation (2.91 kg ha^−1^ and 32%) (Fig. 10).

## Discussion

Acid ions resulted in acid precipitation according to direct pH measurements. The interception of the mixed plantation canopy was the highest (Table 1, Fig. 3 and Fig. 9), which indicated that the mixed plantation canopy had the most significant effect for reducing SO_4_^2−^ and Cl^−^ emissions. This effect can be explained by the mixed plantation conditions from two aspects. First, the net throughfall and stemflow inputs are the combined result of leaching, dry input and uptake in the canopy layer^22^. Second, the properties of the canopy layer were determined, such as the canopy roughness, canopy length, canopy cover and LAI, and the properties of the individual canopy elements were determined, such as the efficiency of leaves for capturing or absorbing gases and particles, and the surface wetness^23^. Because most of the acidic anions were taken up by the canopy layer^18^, the pH values of the throughfall and stemflow were higher than the pH of the bulk precipitation (Table 1). The acidity of the rain reflects the air pollution index^24^, thus, the canopy layer plays a significant role in reducing pollution and acid inputs, although many ions are leached from the canopy layer^24^,^25^.

When the load in the bulk precipitation is higher than the load in the surface runoff, a surface runoff event occurs^26^,^27^. The surface runoff inputs of both ions were lowest in the mixed plantation and highest in the non-forest land (Fig. 4). One cause of the higher surface runoff inputs in the non-forest land compared with the plantations may be the low vegetation coverage in the non-forest land, which results in greater SO_4_^2−^ and Cl^−^ input^27^. Vegetation coverage was the dominant factor controlling the SO_4_^2−^ and Cl^−^ inputs measured in the surface runoff, and the highest inputs of both ions appeared in the non-forest land, followed by cryptomeria, camphor, and mixed plantations (Fig. 4, Fig. 8 and Fig. 9). These results agree with the results of Fenn *et al*.^15^. Likewise, the net inputs of both ions from processing the surface runoff decreased from the mixed plantation to the cryptomeria and camphor plantations when compared with the non-forest land (Fig. 10). Except for the influencing factors, such as surface slope, initial soil moisture content and surface roughness^28^,^29^, the results can be explained by the high vegetation coverage and rich tree species diversity in the mixed plantation. The net inputs in the surface runoff were significantly lower in the three plantations for SO_4_^2−^ and no significant differences were observed for Cl^−^ (Fig. 10). These results are consistent with the current consensus that surface runoff preferentially takes up Cl^−^ rather than SO_4_^2−^ from the cover layer and that Cl^−^ likely enters the soil and results in lower Cl^−^ concentrations in the surface runoff^22^,^26^. All of the pH values in the three plantations increased compared with the non-forest land. This result agrees with the results of other studies in which the cover layer was used to estimate the critical load and good mixed vegetation patterns significantly decreased the acidity of the bulk precipitation^22^,^26^.

The soil layer is often considered the main factor that influences the soil percolating water and accounts for most soil ion input processes^30^. Most ions were leached from the soil layers, which have high ion contents^31^. The highest outputs of SO_4_^2−^ resulted from the mixed plantation, followed by the camphor and cryptomeria plantations (Fig. 9). Several factors affect these processes, and some of these factors are likely the same between the different studied forest plots (soil texture, quantity and the duration of precipitation and abiotic stresses). The factors that affect these process that were different between the studied forest plots include the plant roots, soil aggregate stability and porosity, which were considerably different among the tree species, as well as the tree physiology and potentially the age distribution of the leaves^32^,^33^. Hence, the differences in the exchange between the plants and soils at the four plots potentially resulted from differences in the above-mentioned factors. Furthermore, compared with the non-forest land, the three plantations all intercepted certain amounts of both ions (Fig. 9 and Fig. 10). The pronounced leaching of SO_4_^2−^ and Cl^−^ from the soil layer indicated that the soil properties in the plots were not the same. The pH values of the soil layers significantly increased, and the SO_4_^2−^ and Cl^−^ were higher in the soil percolating water than in the bulk precipitation, throughfall, stemflow and surface runoff. The mean pH of the soil percolating water reached up to 6.13 in the MF (Fig. 8). These results indicated that the pH and acid anion concentrations were not directly related in the soil layer and that the acid load was significantly different between the four soil plots. In addition, most of the SO_4_^2−^ and Cl^−^ ions originated from the soil hydrology itself. Because it was not possible to accurately distinguish and estimate the inputs of SO_4_^2−^ and Cl^−^ in the soil, the uptake and leaching of SO_4_^2−^ and Cl^−^ in the soil layer was potentially overestimated^18^. Because soil inputs were influenced by the stand characteristics, the actual differences in soil exchange among the four plots could differ from the calculated differences^34^.

Input modes (wet or dry) may depend strongly on the local climatology and bulk precipitation. Dry input is dominant over wet input in many countries in northern China^35^,^36^. However, because of the higher precipitation, wet input may be dominant in the rainy area of West China. This finding has been reported for many Asian countries^37^,^38^ and is confirmed by the data presented in this study. Forest ecosystems can intercept SO_4_^2−^ and Cl^−^ from the canopy, cover and soil layers during rainfall^39^, which could be important for reducing atmospheric sulfur and chlorine input. The inputs and outputs in forest ecosystems exhibited different patterns in each layer in the three plantations. The mixed plantation resulted in the highest interception of both ions by the canopy and cover layers but the lowest interception of the ions by the soil layer (Fig. 9 and Fig. 10). The results of Guckland *et al*.^40^ suggested that species-related differences in the intensity of anion cycling between soils and trees contributed to the observed differences in soil acidification among the studied forest plots. Thus, tree species potentially contributed to the differences in the soil properties. In addition, in a common garden test with 14 tree species, including *Tilia cordata, Acer pseudoplatanus, Acer platanoides, Fagus sylvatica* and *Carpinus betulus*, the type of tree species directly influenced the soil properties through variations in litter quantity and chemistry and indirectly influenced the soil properties through the effects of litter on detritivores^41^.

In this study, the net inputs of SO_4_^2−^ and Cl^−^ followed different trends, and significant differences were observed among the three plantations (Fig. 10). The input of both ions was high, which resulted in higher interception of both ions by the forest ecosystem. However, these forests had soils with high critical acritical loads, high rates of acidic input and lower forest pollution saturation^4^. A few studies are available from India where individual species rather than an ecosystem were used to estimate the critical pollutant load^42^,^43^,^44^. However, these studies cannot be used to frame environmental guidelines for preventing acidifying pollution because they do not represent the entire ecosystem or its most sensitive elements. Therefore, urgency necessitates the use of refined insight to determine the direct and indirect effects of SO_4_^2−^ and Cl^−^ precipitation in urban areas and in farmland, grassland, river and lake ecosystems^45^,^46^, particularly because the maintenance of ecosystems and their health is an important prerequisite for biosphere sustainability. Therefore, a “forest filter” can be used to buffer pollution from atmospheric input during rainfall^47^,^48^ to establish a “atmospheric pollutants input – plant – litter – soil – microbial” ecological cycle and to provide a theoretical basis for forest management in polluted areas.

In summary, the three plantations can be used as “forest filters”, and the mixed plantation has the best filtering efficiencies for both SO_4_^2−^ and Cl^−^. The camphor plantation can filter more SO_4_^2−^ than the cryptomeria plantation, but the cryptomeria plantation can filter more Cl^−^ than the camphor plantation. Furthermore, the filtering efficiencies of the three plantations for SO_4_^2−^ are not identical in different layers. In the mixed plantation, the canopy and cover layer have the strongest ability to intercept SO_4_^2−^ while the soil layer has the best ability to intercept SO_4_^2−^ in the cryptomeria plantation. Moreover, the mixed plantation always shows the best filtering ability for Cl^−^ in the different layers. It was impossible to isolate the roles of single stand characteristics in nature. Although the mixed plantation did not show the best filtering effect for both pollutants in every layer, our results suggest that this forest ecosystem has the best filtering ability. In addition, the advantage of the mixed plantation for filtering pollutants could become more effective as the BP increases, especially during heavy rainfall events and the rainy season. In summary, we conclude that the three plantations can significantly change the distribution and transfer of elements (especially the mixed plantation), which suggests that forests with different tree species have the best ability to filter and intercept pollutants.

## Materials and methods

### Study area

Our study was conducted in the rainy area of West China (E102°59′, N29°58′, 667 m.a.s.l.) in Ya’an City, Sichuan, West China. The reserve is located in a transitional area between the Tibetan Plateau and the Sichuan Basin. The mean annual temperature (recorded in a large opening) was 16.1 °C, with monthly average temperatures ranging from 3.7 °C to 29.9 °C^38^. The mean annual precipitation was approximately 1732 mm, with up to 263.5 rainy days. The number of mean annual sunshine days in the city is 1019 h, and the MAS rate is 23%. The soil is classified as acid purple soil with an effective soil thickness (eluvial and illuvial soil layers) of less than 50 cm^38^. The typical vegetation in the region includes camphor (*Cinnamomum camphora*) plantations and cryptomeria (*Cryptomeria fortune).* Study plots where randomly established in the four following types of vegetation communities (Table 3): a camphor plantation, cryptomeria plantation, mixed plantation and area of non-forest land. Four study plots (each 0.16 ha) with different tree species diversities were selected within a radius of approximately 4 km in the south-western area of Sichuan Agricultural University. The selected forest plots were located in a contiguous forest area and were well managed. Each forest plot was assigned to one of the four following tree species diversity levels (DL).

DL1: the tree layer consists of at least 95% *C. camphora*;

DL2: the tree layer consists of at least 90% *C. fortunei* and 6% *C. camphora*;

DL3: the tree layer consists of at least 60% *C. camphora*, 10% *C. fortunei* and 25% other tree species (*Cinnamomum longepaniculatum, Metasequoia glyptostroboides, Broussonetia papyrifera, Rosa rubus*, etc.) and the canopy density of the broad-leaved evergreen tree species is 0.75;

DL4: the non-forest land land is a grassland with herbaceous coverage of only 0.12 (*Ophiopogon chingii, Erigeron acer, Eleusine indica, Dichondra repens, Veronica undulate*, etc.).

### Study design

The following rain water fractions were sampled after each rainfall event: bulk precipitation, throughfall, stemflow, surface runoff and soil percolating water. bulk precipitation was sampled at three sites outside the three plantations located approximately 50 m from the forest edge, and 5 continuously open precipitation collectors were placed at each site. The distances between the selected plantation plots ranged from 200 to 1,000 m. Each fraction was sampled by 15 collectors to reduce sampling effort in each of the forest plot. The collectors were located along five randomly selected 40 m long transects with three collectors along each transect. The three following collector types (CT) were used in each plantation.

CT1: large collector (actual surface collection area of 200 cm × 15 cm), the PVC rectangle groove technique was used to quantify the bulk precipitation and throughfall. CT1 was mounted on a tripod made of wood at a height of 1 m above the ground to avoid splashing mud into the collector.

CT2: small collector (actual surface collection area of 50 cm × 15 cm), the PVC U-shaped groove technique was used to quantify the soil percolating water. CT2 was placed at an angle of nearly 5° to the surface and positioned at a soil depth of 50 cm.

CT3: large bottle collector (actual colleting volume 15 L), the PE bottle technique was used to quantify the surface runoff. A small surface watershed was built (actual surface collection area of 5 m × 5 m) to collect surface runoff.

CT4: small bottle collector (actual colleting volume of 5 L), the PE bottle technique was used to quantify the stemflow. To collect stemflow, the trees were equipped with flexible tubing (garden hose) cut in half longitudinally and fixed tightly around the tree trunks in a steeply sloped upward spiral to avoid overflow. The tubing was stapled to the tree trunk and silicone sealant was applied to seal the collar to the trunk to avoid stemflow losses.

The collectors were placed in March 2011 before the rainy season and removed after collecting the first sample to avoid the influences of the material and humans. Sampling for hydrochemistry was performed on an event basis (n = 15) from April to December 2011 after single rainstorms. Precipitation events of less than 0.3 mm were discarded in consideration of the precipitation intercepted by the canopy layer. Overall, 40 precipitation events were sampled (0.39–39.9 mm rainfall) and analysed to determine the SO_4_^2−^ and Cl^−^ concentrations in the bulk precipitation, throughfall, stemflow, surface runoff and soil percolating water. In addition, because several collectors were destroyed due to the continuous influence of rain, samples were not collected from the five bulk precipitation events during the observation period. We analysed each collected sample individually to determine the heterogeneity of the SO_4_^2−^ and Cl^−^ fluxes and the factors controlling the SO_4_^2−^ and Cl^−^ fluxes. Thus, the samples were not combined for each event, tree size class or plot.

### Chemical analyses

For chemical analyses, the water from the bulk precipitation collectors at each site was pooled, which resulted in 15 replicate samples per day for the bulk precipitation. The water from the 15 bulk precipitation collectors at each site was pooled, which resulted in 15 replicate samples per forest plot and date for the throughfall. All stemflow samples were analysed chemically. The volumes of stemflow for each plot were calculated using species-specific regressions between the diameter at breast height (DBH), the stemflow volumes from each measurement period, the total number of stems and the total DBH of the trees on each studied plots. When no persistent correlation existed between the DBH and stemflow for a certain species, an average stemflow value was calculated for all of the measured trees of the considered species. The surface runoff was monitored in each plot from 15 replicates to determine the mass fraction of elements and the export of surface runoff by using the runoff plot method^8^. Representative soil percolating water samples were collected from the 15 replicates at a depth of 50 cm because 90% of the root biomass was present^49^, the soil constituents at reflected the soil parent material and the soils were free from wind-blown materials at this depth^8^. The concentrations of all the investigated chemical compounds were multiplied by the water volumes of each sample to provide the quantities of each chemical compound per sample. This concentration was then multiplied by the volume of water per plot and upscaled to one hectare to provide quantities of each chemical compound per hectare (kg ha^−1^) in the bulk precipitation, throughfall, stemflow, surface runoff and soil percolating water.

Bulk precipitation, throughfall, stemflow, surface runoff and soil percolating water samples from each collector were pooled (on a volume-weighted (vw) basis) for chemical analysis. The retrieved samples were immediately transported to the laboratory, and pH measurements were performed within 24 h after sampling by using a Systronics digital pH meter. Before proceeding with further analysis, the samples were filtered using filter paper with a 0.45-μm pore size (Millipore™) and refrigerated at 4 °C. All samples were analysed within a week of their collection, sub-samples were analysed calorimetrically for SO_4_^2−^ and Cl^−^, and all samples were analysed using standard methods (APHA, 2005). The SO_4_^2−^ and Cl^−^ ions were measured using a spectrophotometer and the barium chromate and ferrithiocyanate method (ultraviolet-visible spectrophotometry, UV-VIS), respectively.

### Calculations

The volume-weighted mean concentrations for bulk precipitation (BP), throughfall (TF), stemflow (SF), surface runoff (SR) and soil percolating water (SP) were calculated by weighting each of the samples. The fluxes of BP, TF, SF, SR and SP were calculated as the products of the volume-weighted mean concentrations from the precipitation volumes of the 9-month period and expressed on an annual basis.

*Stemflow* (SF, Eq. (1)): The SF in each plantation was calculated by multiplying the number of tree (*Tn*) stems per square metre (*S*) in the respective size classes (*Sn*, 5–10 cm, 10–15 cm, 15–20 cm, 20–25 cm, >25 cm DBH) in a plot^50^.


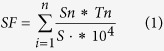


*Volume-weighted mean concentrations* (VWM, Eq. (2)): The VWM concentrations in the BP, TF, SF, SR and SP and in the bulk deposition collector were used to express the solute concentration during the study period^51^, where *Ci* represents the concentration of a specific solute and *Vi* is the volume during this event.


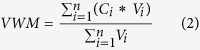


*Annual fluxes* (AF) per tree (Eq. (3)): The AFs per tree were calculated by multiplying the volume-weighted mean values of each forest BP, TF, SF, SR and SP (VWM) by the volume ratio, which equalled the BP volume of the respective tree during the sampled rain event (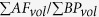) multiplied the annual BP volume. The annual solute fluxes in the BP where multiplied using the quotient of the basal area to plot the area, and these values were subtracted from the BP values.


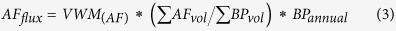


*Net annual fluxes* (NAF, (Eq. (4)): The NAFs were summed over canopy sizes to calculate the annual net fluxes of the respective camphor plantation, cryptomeria plantation and mixed plantation plots.


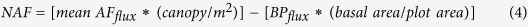


### Statistical analyses

The objectives of this study were to evaluate the differences in the interception input, total input, canopy exchange and stand input between the forest types and along the tree species diversity gradient. Thus, the 9-month means of the interception input, total input, canopy exchange and stand input of each investigated chemical compound were used as dependent variables in analysis of variance (ANOVA), and forest type was used as the explaining variable. The differences between the forest types were considered significant if they exceeded the least significant difference (LSD) that was computed for each pair of DLs (p < 0.05). After conducting pairwise comparisons using multiple ANOVA, the responses of the variables (BP, TF, SF, SR and SP) across the position properties to the input time were evaluated using exponential regression or non-parametric LOESS regression with 95% confidence intervals (95% CI). After verifying the general ANOVA hypothesis, detailed posthoc mean comparisons were performed to determine the significant differences of the SO_4_^2−^ and Cl^−^ variables (all the measurements and calculations) among the positions during each rainfall event using Tukey’s honestly significant difference (HSD). The homogeneity of the variances was tested using Levene’s test. Any data sets failing this test were log-transformed before further analysis to help satisfy the requirements of variance homogeneity. The effects of forest type on the SO_4_^2−^ and Cl^−^ input were evaluated using ANOVA. All analyses were performed in the Statistical Product and Service Solutions programme (SPSS version 21.0).

## Additional Information

**How to cite this article**: Zhao, H. *et al*. Mixed forest plantations can efficiently filter rainfall deposits of sulfur and chlorine in Western China. *Sci. Rep.* **7**, 41680; doi: 10.1038/srep41680 (2017).

**Publisher's note:** Springer Nature remains neutral with regard to jurisdictional claims in published maps and institutional affiliations.

## Figures and Tables

**Figure 1:**
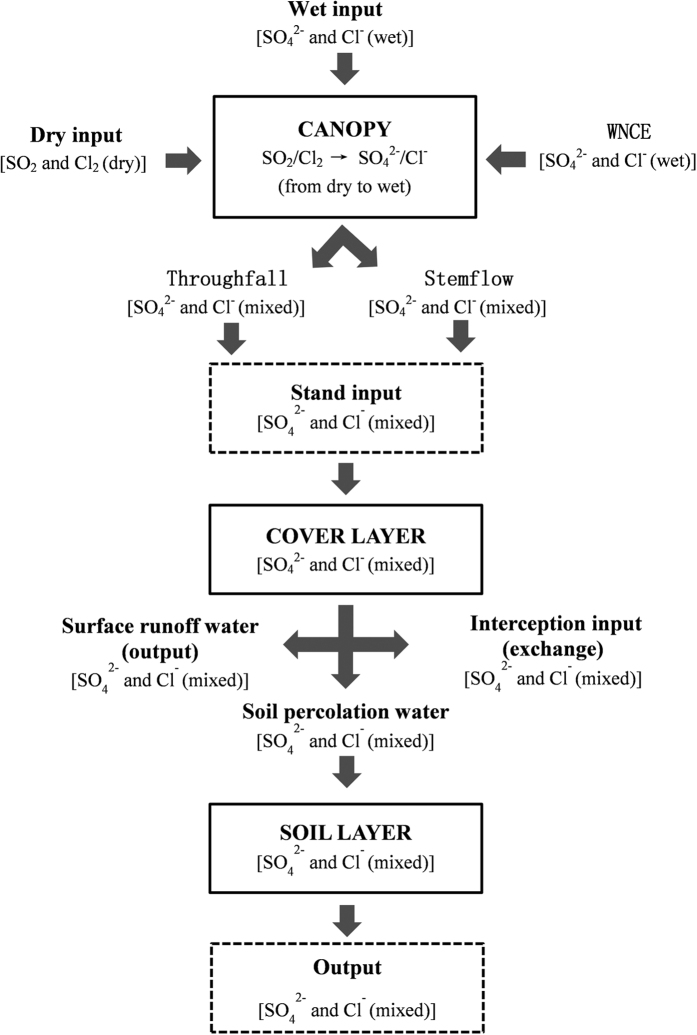
The inputs and outputs of sulfur and chlorine throughout the forest ecosystem. WNCE: Water Net Canopy Exchange.

**Table 1:** Relationship between the three plantations (TF, SF and pH values) and BP.

Plantation	Species	Index	Fitting equation	Parameters		n	*R*^2^	*P*
				*a*	*b*			
CCP	TF	SO_4_^2−^	TF = *aP* + *b*	−0.078	4.341	40	0.850	0.000
		Cl^−^	TF = *aP* + *b*	−0.008	0.505	40	0.889	0.000
		pH	TF = *aP* + *b*	0.044	5.191	40	0.869	0.000
	SF	SO_4_^2−^	SF = *aP* + *b*	−0.058	4.054	23	0.855	0.000
		Cl^−^	SF = *aP* + *b*	−0.006	0.482	23	0.877	0.000
		pH	SF = *aP* + *b*	0.041	5.132	23	0.947	0.000
CFP	TF	SO_4_^2−^	TF = *aP* + *b*	−0.071	4.290	40	0.861	0.000
		Cl^−^	TF = *aP* + *b*	−0.007	0.500	40	0.944	0.000
		pH	TF = *aP* + *b*	0.040	5.173	40	0.916	0.000
	SF	SO_4_^2−^	SF = *aP* + *b*	−0.049	4.015	16	0.982	0.000
		Cl^−^	SF = *aP* + *b*	−0.006	0.491	16	0.953	0.000
		pH	SF = *aP* + *b*	0.035	5.192	16	0.975	0.000
MP	TF	SO_4_^2−^	TF = *aP* + *b*	−0.079	4.291	40	0.802	0.000
		Cl^−^	TF = *aP* + *b*	−0.008	0.504	40	0.839	0.000
		pH	TF = *aP* + *b*	0.056	5.106	40	0.876	0.000
	SF	SO_4_^2−^	SF = *aP* + *b*	−0.071	4.289	33	0.823	0.000
		Cl^−^	SF = *aP* + *b*	−0.008	0.505	33	0.877	0.000
		pH	SF = *aP* + *b*	0.053	4.999	33	0.930	0.000

BP: bulk precipitation.

CCP: Camphor (*Cinnamomum camphora*) plantation.

CFP: Cryptomeria (*Cryptomeria fortunei*) plantation.

MP: mixed plantation.

SF: stemflow.

TF: throughfall.

**Table 2:** The mean concentrations of pollutants in BP, TF, SF, SR and SP under the four types of growth conditions.

Species	Plot	BP	TF	SF	SR	SP
SO_4_^2−^(mg·L^−1^)	CCP	3.474 ± 0.667c	3.381 ± 0.957c	3.102 ± 0.683c	5.509 ± 0.486b	9.991 ± 1.890a
	CFP	3.474 ± 0.667c	3.411 ± 0.870c	3.089 ± 0.530c	5.359 ± 0.769b	9.326 ± 1.971a
	MP	3.474 ± 0.667c	3.312 ± 1.004c	3.279 ± 0.868c	4.719 ± 0.302b	10.820 ± 1.823a
	NFL	3.474 ± 0.667c	—	—	5.775 ± 0.906b	8.390 ± 1.635a
Cl^−^(mg·L^−1^)	CCP	0.418 ± 0.075c	0.406 ± 0.097c	0.376 ± 0.075c	0.642 ± 0.110b	1.047 ± 0.248a
	CFP	0.418 ± 0.075c	0.414 ± 0.082c	0.378 ± 0.065c	0.630 ± 0.130b	0.972 ± 0.227a
	MP	0.418 ± 0.075c	0.401 ± 0.104c	0.398 ± 0.089c	0.639 ± 0.082b	1.199 ± 0.242a
	NFL	0.418 ± 0.075c	—	—	0.715 ± 0.188b	0.883 ± 0.216a

Note: Letters a, b and c represent each index with a different degree of significant difference.

BP: bulk precipitation.

CCP: Camphor (*Cinnamomum camphora*) plantation.

CFP: Cryptomeria (*Cryptomeria fortunei*) plantation.

CP: concentrations of pollutants.

MP: mixed plantation.

NFL: non-forest land.

SF: stemflow.

SP: soil percolating water.

SR: surface runoff water.

TF: throughfall.

**Table 3:** The characteristics of the vegetation of the sample plot.

Vegetation types	Tree species (DL)	Slope	Strain density (plant/hm^2^)	Canopy density	Average DBH (cm)	Tree height (m)	Forest reserves (m^3^)	Forest age (year)
CCP	DL1	27.6°	960	0.92	25.40	19	74.255	18
CFP	DL2	19.8°	577	0.71	16.86	16	51.540	17
MP	DL3	20.0°	719	0.87	16.78	18	58.686	18
NFL	DL4	21.3°	—	—	—	—	—	—

CCP: Camphor (*Cinnamomum camphora*) plantation.

CFP: Cryptomeria (*Cryptomeria fortunei*) plantation.

DBH: diameter breast height.

DL: diversity level.

MP: mixed plantation.

NFL: non-forest land.
